# Genetic diversity, population structure, and traditional culture of *Camellia reticulata*

**DOI:** 10.1002/ece3.3340

**Published:** 2017-09-21

**Authors:** Tong Xin, Weijuan Huang, Jan De Riek, Shuang Zhang, Selena Ahmed, Johan Van Huylenbroeck, Chunlin Long

**Affiliations:** ^1^ College of Life and Environmental Sciences Minzu University of China Beijing China; ^2^ Plant Sciences Unit Institute for Agricultural and Fisheries Research Melle Belgium; ^3^ Department of Health & Human Development Montana State University Bozeman MT USA; ^4^ Kunming Institute of Botany Chinese Academy of Sciences Kunming China

**Keywords:** *Camellia reticulata*, ethnobotany, genetic diversity conservation, traditional culture

## Abstract

*Camellia reticulata* is an arbor tree that has been cultivated in southwestern China by various sociolinguistic groups for esthetic purposes as well as to derive an edible seed oil. This study examined the influence of management, socio‐economic factors, and religion on the genetic diversity patterns of *Camellia reticulata* utilizing a combination of ethnobotanical and molecular genetic approaches. Semi‐structured interviews and key informant interviews were carried out with local communities in China's Yunnan Province. We collected plant material (*n* = 190 individuals) from five populations at study sites using single‐dose AFLP markers in order to access the genetic diversity within and between populations. A total of 387 DNA fragments were produced by four AFLP primer sets. All DNA fragments were found to be polymorphic (100%). A relatively high level of genetic diversity was revealed in *C. reticulata* samples at both the species (*H*_sp_ = 0.3397, *I*_sp_ = 0.5236) and population (percentage of polymorphic loci = 85.63%, *H*_pop_ = 0.2937, *I*_pop_ = 0.4421) levels. Findings further revealed a relatively high degree of genetic diversity within *C. reticulata* populations (Analysis of Molecular Variance = 96.31%). The higher genetic diversity within populations than among populations of *C. reticulata* from different geographies is likely due to the cultural and social influences associated with its long cultivation history for esthetic and culinary purposes by diverse sociolinguistic groups. This study highlights the influence of human management, socio‐economic factors, and other cultural variables on the genetic and morphological diversity of *C. reticulata* at a regional level. Findings emphasize the important role of traditional culture on the conservation and utilization of plant genetic diversity.

## 1 INTRODUCTION

Biodiversity is fundamental for life on earth by supporting the health of ecosystems (Mace et al., 2014). However, rapid economic development, social and cultural shifts, habitat loss, overexploitation of resources, climate change, and pollution are posing serious threats to biodiversity (Cardinale et al., 2012) including eliminating the plant genetic resources that support life on earth (Garcia, Gabeza, Rahbek, & Araujo, 2014; Rands et al., 2010). Effective conservation of biodiversity is called for to support the maintenance of ecosystem processes that ultimately sustain human life (Khairo, 2016).

Traditional cultural practices and local ecological knowledge of smallholder farmers and indigenous communities that has accumulated over generations have been widely recognized to contribute to biodiversity conservation (Bohn, Diemont, Gibbs, Stehman, & Vega, 2014; Shen et al., 2012) and may potentially be tapped for addressing the rapid loss of biodiversity around the world. Previous studies have acknowledged the role of traditional knowledge and culture practices of smallholder farmers and indigenous communities for biodiversity conservation at the species, genetic, ecosystem, and landscape levels (Altieri, 2004). Many traditional management practices, customs, and beliefs have been reported to contribute to biodiversity protection including seed exchange systems (Labeyrie, Thomas, Muthamia, & Leclerc, 2016), marriage exchanges (Delêtre, McKey, & Hodkinson, 2011), religious rituals (Mazumdar & Mazumdar, 2012), and dietary traditions (Penafiel, Lachat, Espinel, Van Damme, & Kolsteren, 2011). These practices, customs, and beliefs have been linked to preserving crop landraces (Jackson, Pascual, & Hodgkin, 2007), old trees (Salick et al., 2007), and economic plants (Liu et al., 2014) including those with esthetic, food, and medicinal values (Begum et al., 2014).

*Camellia reticulata* Lindl. (Theaceae), an evergreen arbor (Liu & Gu, 2011) known as “Yunnan *shan‐cha*” in Chinese, has a long history of being cultivated by various sociolinguistic groups of China's Yunnan Province including the Yi, Bai, Naxi, Hui, Miao, and Lisu for its ornamental, horticultural, and cultural values as well as for the oil derived from its seed (Xin et al., 2015). The Bai ethnic group cultivates *C. reticulata* in their gardens as a symbol of gentility, and the Yi pay special respect to camellias while communities in Tengchong extract an edible oil from the seeds (Wang & Ruan, 2012). *C. reticulata* is naturally distributed in Yunnan, southwest Sichuan and Guizhou Provinces of China (Ming, 2000). Morphologically, *C. reticulata* has attractive large flowers (7–18 cm in width) with bright red or pink petals (Li, Hashimoto, Shimizu, & Sakata, 2007) that have a long blossoming season in winter.

*Camellia reticulata* is a perennial, outcrossing, and heterogenous double ploidy species (2*n* = 30) (Ming, 2000). The cultivation of *C. reticulata* dates back to the Sui and Tang dynasties (~600 AD). This species has been widely described in classical literature including poems and inscriptions (Wang et al., 2011). Camellias are widely cultivated in Buddhist temples and offered to the Buddha (Xin et al., 2015). The British botanist J. Lindley identified and named *Camellia reticulata* in the Botanical Register in 1827 and introduced this species to Europe (Ming, 2000). By the 1950s, *C. reticulata* was found in the gardens of western countries and continues to be desired as an ornamental including by the potted flower industry (Hattan et al., 2016).

Managers of *C. reticulata* have bred this resource with other *Camellia* species to produce colorful cultivars with different colors and blossom periods (Zhou et al., 2014). During the process of polyploidization and hybridization, other *Camellia* species have likely contributed to the genetic diversity of *C. reticulata* (Hong, 2010; Wang & Ruan, 2012; Yuan, Cornille, Giraud, Cheng, & Hu, 2014) and resulted in over 500 cultivars and hybrids (Chen, Wang, Xia, Xu, & Pei, 2005). However, with rapid environmental and socio‐economic change as well as a trend toward monocultures and reduced genetic diversity (Barrett, Travis, & Dasgupta, 2011; Cardinale et al., 2012), it is becoming important to understand the current status of plant genetic diversity of *C. reticulata* (Wang, Chiang, Roux, Hao, & Ge, 2007). While numerous studies have reported on the importance of traditional management practices for biodiversity conservation, the complex interaction between cultural practices and genetic diversity of *C. reticulata* has not been studied (Hong, 2010).

With the development of biotechnology, molecular marker analysis has been found to effectively assess genetic diversity (Thomas, Vijayan, Joshi, Joseph, & Raj, 2006). AFLP analysis has been widely used to characterize natural populations and breeding gene pools (De Riek, De Cock, Smulders, & Nybom, 2013; Roldàn‐Ruiz, Dendauw, Van Bockstaele, Depicker, & De Loose, 2000) and is considered highly informative for studying genetic diversity and phylogenetic relationships by identifying variations with high resolution at the level of an individual's DNA (Agarwal, Shrivastava, & Padh, 2008; Costa, Pereira, Garrido, Tavares‐de‐Sousa, & Espinosa, 2016).

In this study, we integrated ethnobotanical and molecular genetic approaches to examine the influence of cultural factors on the conservation of genetic diversity of *C. reticulata*. Semi‐structured and key informant interviews were carried out with managers of *C. reticulata* in central and western parts of Yunnan Province of China. Samples from five distinct populations of *C. reticulata* were collected from Yunnan in order to analyze the genetic diversity and population structure using AFLP analysis. It is expected that the genetic diversity of *C. reticulata* has been influenced by the cultural practices of various sociolinguistic groups who have managed, cultivated, and conserved this tree resource.

## 2 MATERIALS AND METHODS

### 2.1 Ethnobotanical survey and plant collections

**Figure 1:**
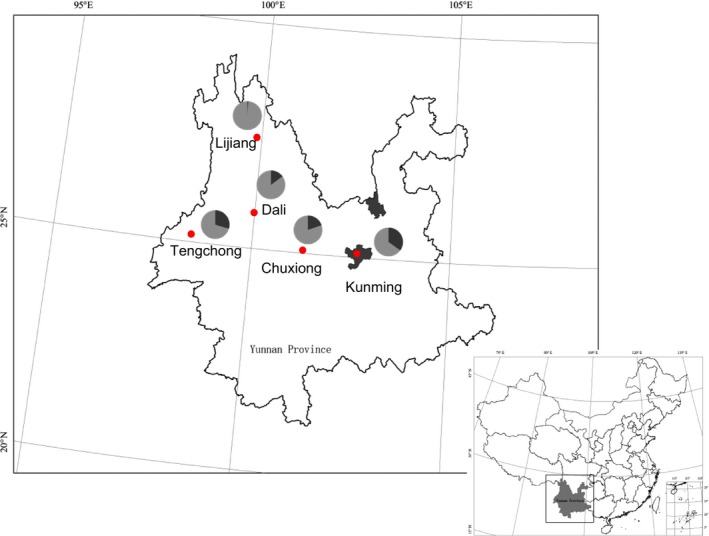
Sample locations of *Camellia reticulata* in Yunnan Province, China (the dark black part of the pie above represents the proportion of different sample size: 66 samples from Kunming [34.9%], 56 samples from Tengchong [29.6%], 37 samples from Lijiang [19.6%], 28 samples from Dali [14.8%], and two samples from Chuxiong [1.1%])

Research was carried out in the main distribution and production areas of *C. reticulata*. This includes locations in central and western parts of Yunnan Province in five prefectures including the following: Kunming, Chuxiong, Dali, Tengchong (in Baoshao City), and Lijiang (Figure 1). Ethnobotanical methods including semi‐structured interviews and key informant interviews were conducted to understand the history, culture, use, local names, and number of cultivars of *C. reticulata*. Informants were randomly selected from local community members in different socio‐linguistic groups (including the Yi, Bai, Dai, et al. who have their own unique language and culture) at the study sites who were willing to participate in surveys. A total of 120 informants participated in this study including 38 from Kunming, 20 from Chuxiong, 25 from Dali, 26 from Tengchong, and 11 from Lijiang. In addition, we examined relevant literature regarding beliefs, traditional knowledge, and customs on *C. reticulata* at the study sites.

A total of 190 individuals from five populations were sampled from the five study sites. Their local name and the morphological characteristics including flower color, flower type, and blooming period were recorded according to ethnobotanical survey as well as plant database research (see Appendix S1).

### 2.2 AFLP analysis of genetic diversity

Approximately 30–40 individuals of *C. reticulata* were collected from each population. From each sampled plant, young, healthy leaves were collected, silica‐dried, and stored at −20°C for laboratory analysis of genetic diversity. Genomic DNA was extracted from 15 to 30 mg dried‐leaf samples following modified CTAB method (Doyle & Doyle, 1990). AFLP was performed according to Vos et al. (1995). Each diluted DNA sample was digested with *Eco*RI and *Mse*I and ligated to specific adapters using T4 DNA ligase. *Eco*RI + A and *Mse*I + C primers were used for preamplification (Table S1). PCR reactions were performed in a GeneAmp PCR system 9700 (ABI, USA). The preamplification program consisted of 25 cycles of 30 s at 94°C, 1 min at 56°C, and 1 min at 72°C. Evaluation of the restriction digest, adapter ligation, and the preamplification occurred on a 1.5% TAE buffered agarose gel, using λ *Pst* as size reference (Tables S2 and S3). Selective amplification was performed with four *Eco*RI/*Mse*I primer combinations with six selective bases (fluorescent labeling of the *Eco*RI primers): *Eco*RI‐ACT/*Mse*I‐CAC (PC1), *Eco*RI‐ACC/*Mse*I‐CAA (PC2), *Eco*RI‐ACC/*Mse*I‐CTC (PC3), and *Eco*RI‐ACG/*Mse*I‐CTC (PC4). The amplification program consisted of 2 min at 94°C, 30 s at 65°C, 2 min at 72°C, followed by 8 cycles of 1 s at 94°C, 30 s at 64°C, and 2 min at 72°C (the temperature decreased after each cycle with 1°C, from 64°C to 56°C) and finally 23 cycles of 1 s at 94°C, 30 s at 56°C and 2 min at 72°C (Table S4). AFLP fragments were separated on a 3130xl Genetic Analyzer (Applied Biosystems). GeneMapper v4.0 (Applied Biosystems) was used to record the signal peak height, the signal area, and the fragment size. The data were exported to access, and a scoring table was generated as a starting point for the data analysis. The bands were scored as either present (1) or absent (0) across all loci (see Appendix S2).

### 2.3 Data analysis

Genetic diversity parameters were generated using the program GenALEx v6.5 (Peakall & Smouse, 2012) and POPGEN32 v3.01 (Yeh, Yang, Boyle, Ye, & Xiyan, 2000) in order to provide information on the percentage of polymorphic loci (*P*), Nei's Gene Diversity Index (*H*; Nei, 1973) and Shannon's Information Index (*I*; Frank, Furst, Koschke, Witt, & Makeschin, 2013).

Nei's genetic distances between populations (Nei, 1978) (also generated with POPGEN32) were used to construct unweighted pair group method arithmetic average (UPGMA) dendrograms in NTSYSpc 2.11 after running 100 replicates (McKinnon, Mori, Blackman, & Lior, 2004). Genetic diversity (*H*) was calculated at two levels: the average diversity within populations (*H*_pop_) and the total diversity (*H*_t_). The proportion of diversity among populations was estimated using the following equation: (*H*_t_−*H*_pop_)/*H*_t_. An analysis of molecular variance (AMOVA) was performed using Arlequin (Schneider, Roessli, & Excoffier, 2000) and analyzed from both among populations and within populations. The UPGMA dendrogram of populations based on AFLP fingerprints was drawn based on pairwise similarities using software TFPGA (Miller, 1997).

To further elucidate relationships among all populations, a model‐based cluster analysis was performed using the program STRUCTURE v2.3.1 (Raj, Stephens, & Pritchard, 2014). STRUCTURE was run 20 times and was carried out by setting the number of clusters (*K*) from 1 to 14. The optimum number of clusters (*K*) was processed and identified by STRUCTURE HARVESTER through comparing log probabilities of data for each value of *K* (Earl & Vonholdt, 2012; Stephens & Pritchard, 2003). The output of structure analyses was visualized using the software CLUMPP v1.1.2 (Kopelman, Mayzel, Jakobsson, Rosenberg, & Mayrose, 2015) and DISTRUCT v1.1 (Rosenberg, 2016). Data were input in an Excel spreadsheet in order to carry out statistical analysis and to make figures depicting genetic diversity and cultivar diversity of *C. reticulata*.

## 3 RESULTS

### 3.1 Ethnobotanical survey *Camellia reticulata*

Results of ethnobotanical survey have been published by the author (Xin et al., 2015) that highlighted the impact of traditional culture on the conservation of *C. reticulata*. In this survey, we found that there are 206 ancient trees of *C. reticulata* in Kunming, with 28% of them well maintained in old Buddhist temples including the Panlong temple and Huating Temple. In Chuxiong, 26 of the 72 old *C. reticulata* trees are maintained in Buddhist temples. In Lijiang, there are two different cultivars of old *C. reticulata* trees that have existed for over 500 years and are entitled as “the king of camellia.” Furthermore, there is an old *C. reticulata* in Dali that has grown for about 400 years in the highest altitude of 2,750 m among all *Camellia* species.

### 3.2 Genetic diversity of *Camellia reticulata*

A total of 387 AFLP unambiguous bands (100%) with a gene size ranging from 50 bp to 455 bp were obtained from the 190 plant samples collected for this study with the four *Eco*RI/*Mse*I primer sets (Table 1). The observed effective number of alleles (*N*_e_), Nei's Gene Diversity (*H*), Shannon's Information Index (*I*), total genetic diversity (*H*_t_), genetic diversity within populations (*H*_s_), and gene differentiation coefficient (*G*_ST_) is shown in Table 1. From the data displayed in Table 1, the PC1, PC2, PC3, and PC4 primer sets obtained 145, 80, 71, and 91 polymorphic bands, respectively. Of the obtained polymorphic bands, the highest genetic variation was detected in PC2 (*N*_e_ = 1.6041, *H* = 0.3578, *H*_t_ = 0.3483, and *H*_s_ = 0.3285), whereas PC4 showed the lowest level of genetic diversity (*N*_e_ = 1.5414, *H* = 0.3320, *H*_t_ = 0.3210, and *H*_s_ = 0.3027).

**Table 1:** Polymorphism characteristics of four AFLP primer combinations used in this study

Primer code	Primer sets	Polymorphic bands	*N*_e_*	H*	I*	*H*_t_	*H*_s_	*G*_ST_	*N*_m_
PC1	E‐ACT/M‐CAC	145	1.5919	0.3514	0.5289	0.3490	0.3316	0.0475	23.5509
PC2	E‐ACC/M‐CAA	80	1.6041	0.3578	0.5369	0.3483	0.3285	0.0536	15.2925
PC3	E3‐ACC/M‐CTC	71	1.5775	0.3449	0.5215	0.3350	0.3204	0.0411	23.2286
PC4	E‐ACG/M‐CTC	91	1.5414	0.3320	0.5072	0.3210	0.3027	0.0520	17.4138
Total		387	1.5787	0.3465	0.5236	0.3397	0.3221	0.0487	20.3415

*N*_e_, Effective number of alleles; *H*, Nei's (1973) gene diversity; *I*, Shannon's Information index; *H*_t_, total genetic diversity; *H*_s_, genetic diversity within populations; *G*_ST_, gene differentiation coefficient; *N*_m_, number of migrants.

For the five *C. reticulata* populations at our five study sites in Yunnan, the total percentage of polymorphic loci (PPL), *N*_e_, *H,* and *I* were 85.63%, 1.4917, 0.2937, and 0.4421, respectively, shown in Table 2. Among the five different populations, PPL ranged from 100% to 28.94%, *H* and *I* varied from 0.3509 to 0.1199 and from 0.5227 to 0.1750, separately. The highest value of PPL (100%) was detected in the Kunming population with values of *H* = 0.3402 and *I* = 0.5145. The lowest value of PPL (28.94%) was found in Chuxiong population with the lowest values of *N*_e_ = 1.2046, *H* = 0.1199, and *I* = 0.1750. Compared to Kunming, Lijiang possessed a lower percentage of polymorphism loci, but had the highest values of *N*_*e*_ = 1.5895, *H* = 0.3509, and *I* = 0.5227.

**Table 2:** Genetic diversity of the five studied populations of *C. reticulata*

Population	PPL (%)	*N*_e_	*H*	*I*
Kunming	100	1.5695	0.3402	0.5145
Dali	99.74	1.5482	0.3298	0.5006
Lijiang	99.74	1.5895	0.3509	0.5227
Tengchong	99.74	1.5467	0.3277	0.4979
Chuxiong	28.94	1.2046	0.1199	0.1750
Total	85.63	1.4917	0.2937	0.4421

PPL, Percentage of polymorphic loci; *C. reticulata, Camellia reticulata*.

### 3.3 Genetic differentiation within and among populations

Total gene diversity (*H*_t_ = 0.3483) was primarily distributed within populations (*H*_s_ = 0.3285) on the basis of the genetic variation detected by PC2 as shown in Table 1. With a low *G*_ST_ (0.0536), findings indicate that relatively low level genetic differentiation exists between the five populations; rather, the majority of genetic variation is found within populations. Based on the *G*_ST_ value, the estimated number of migrants (*N*_m_) was 15.2925 and indicates that the rate of gene flow is high enough for transferring genetic diversity among populations.

Analysis of molecular variance (AMOVA) on genetic differentiation among and within populations of *C. reticulata* was conducted and is shown in Table 3. Findings from AMOVA revealed that 96.31% of the total genetic variations was contributed by differences within populations (*p *< .001), which was notably and significantly higher than that among populations (only 3.96% of total genetic variation was due to difference between populations).

**Table 3:** Analysis of molecular variance (AMOVA) within/among *C. reticulata* populations based on AFLP data

Source of variation	*df*	SSD	MSD	Variance component	Total variance (%)	*p*‐Value
Among populations	4	715.7077	178.927	2.9541	3.69	<.0010
Within populations	189	14,177.1812	77.050	77.0499	96.31	<.0010
Total	193	14,892.8889	255.977	80.004	100	

*df*, degrees of freedom; SSD, sum of squared deviation; MSD, mean squared deviation; *p*‐Value, Significance tests after 1,000 random permutation; *C. reticulata, Camellia reticulata*.

### 3.4 Genetic relationships and geographic distances

The genetic distances among all five populations (Kunming, KM; Lijiang, LJ; Chuxiong, CX; Dali, DL; Tengchong, TC) of *C. reticulata* were calculated using TFPGA Software v1.3 and are shown in Table 4. Variation was found in the genetic distance among populations ranging from 0.0077 (KM vs. CX) to 0.0364 (DL vs. LJ) while the geographic distance among populations ranged from 631.5 km to 104.1 km. The highest genetic distance value was 0.361 between KM and LJ with the longest geographic distance 514.5 km, while the lowest genetic distance value 0.0113 was between populations KM and DL with the geographic distance 330.1 km.

**Table 4:** Geographic distance (km) (above right diagonal) and genetic distance (below left diagonal) among populations of *C. reticulata* based on AFLP analysis

Pop ID	Kunming	Dali	Chuxiong	Tengchong	Lijiang
Kunming (KM)		330.1	148.7	631.5	514.5
Dali (DL)	0.0113		191	321.7	210.6
Chuxiong (CX)	0.0077	0.0123		492	104.1
Tengchong (TC)	0.0189	0.03	0.0167		550.1
Lijiang (LJ)	0.0361	0.0364	0.0308	0.034	

*C. reticulata, Camellia reticulata*.

The correlation analysis between genetic distances and geographic distances among the five populations is presented in Fig. S1. Findings indicate that there was significant correlation between genetic distances and geographic distances among the five populations of *C. reticulata* (*p* = .035, *p* < .05). However, an *R* ^2^ value of 0.037 indicated that about 97% of the variation is not explained by the distance between population pairs, but may be caused by other factors such as hybridization by human cultivation practices.

**Figure 2:**
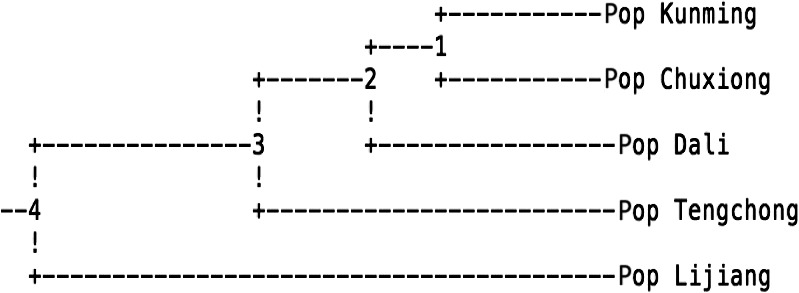
UPGMA dendrogram of *Camellia reticulata* population based on Nei's genetic distance

The UPGMA dendrogram based on Nei's genetic distances that was constructed through POPGENE32 v1.32 (Figure 2) clustered all five populations into different groups. Figure 2 shows that there is no significant correspondence between the genetic structure and geographical origin of *C. reticulata* individuals.

### 3.5 Morphological structure of *C. reticulata* and its correspondence with geographical origin

Morphological findings of the collected 179 *C. reticulata* samples found that flower color is mainly peach pink (79.47% of individuals), pink (4.74%), red (8.95%), white (3.16%), and multicolor (3.68%). The flower type of individuals primarily contained double (73.16% of individuals), semi‐double (13.68%), and single flower type (13.16%). The blooming period of individuals was found to be either early (42.11% of individuals), middle (49.47%), and late (8.42%) (see Appendix S1).

**Figure 3:**
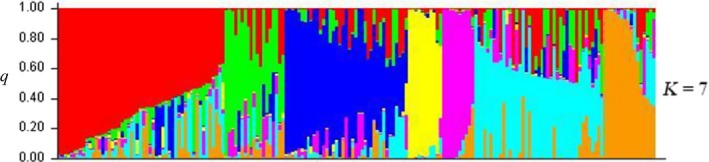
Inference of population morphological structure displayed using STRUCTURE software v2.3.1, STRUCTURE clustering results for *K* = 7 (the red areas stand for cultivars with semi‐double, semi‐double to double, and double red petals mostly from Tengchong. The light blue areas stand for cultivars with single and single to semi‐double red petals as well as the combination of other types. Many traditional cultivars including *Pumencha* and *Luchengchun* are assembled here. The yellow areas collect all 11 cultivars of *Camellia reticulata* from East China. The green areas stand for modern cultivars mostly collected from Kunming.)

Evidence of the population structure of *C. reticulata* and the distribution of flower characteristics was strengthened by multiple models implemented in the program STRUCTURE v2.3.1 (Figs S2 and S3). The optimum number of clusters (*K*) was processed and identified by STRUCTURE, which was *K* = 7 (Figure 3). The populations inferred by STRUCTURE for *K* = 7 were able to distinguish flower characteristics including flower color and flower type. In Figure 3, the red areas represent cultivars that have semi‐double, semi‐double to double, and double red petals; these individuals are mostly from Tengchong. The light blue areas represent cultivars that have single and single to semi‐double red petals as well as the combination of other types. Many traditional *C. reticulata* cultivars including *Pumencha* and *Luchengchun* are assembled within this group. The green areas represent cultivars mostly collected from Kunming.

Multiple flower characteristics of *C. reticulata* were differentiated through statistical analyses including flower type, flower color, and blooming period. Twelve representative flower characteristics of *C. reticulata* were extracted using SPSS's Component Matrix according to Initial Eigenvalues over 1% (Table S5). About 88.60% of flower data of all samples were covered by these 12 flower characteristics as shown in Table 5. Double‐pink‐early maturity (20%) is the most common flower pattern of *C. reticulata*, followed by semi‐double‐pink‐early (17.37%) and double red‐middle (14.21%) (Table 5).

**Table 5:** Results of 12 representative types of *C. reticulata* by eigenvalue extraction according to their flower features

Code	Flower features			Ratio (%)
	Form	Color	Maturity	
1	Double	Pink	Early	20
2	Semi‐double	Pink	Early	17.37
3	Double	Red	Medium	14.21
4	Double	Pink	Medium	12.63
5	Semi‐double	Pink	Medium	10
6	Double	Red	Late	5.26
7	Double	Pink	Early	5.26
8	Semi‐double	White	Early	2.63
9	Semi‐double	White	Medium	2.11
10	Double	White	Late	1.58
11	Double	Pink	Late	1.58
12	Semi‐double	Pink	Late	1.10

*C. reticulata, Camellia reticulata*.

Correspondence between the morphological structure and geographical origin is presented in Figure 5. Peach pink is the main flower color for all the five sites. Double flower type is the most type for all sites except for Tengchong with a much higher number of single type. Early and middle blooming period are the two main blooming periods for all sites except for Dali that has fewer varieties with an early blooming period. Regardless of the flower color, flower type, or blooming period, Kunming was found to have the most amount of *C. reticulata* varieties compared to the other four sites. Therefore, morphological structure seems not to have influence geographical distribution and distances.

### 3.6 Correlation analysis of genetic structure and morphological structure

**Figure 4:**
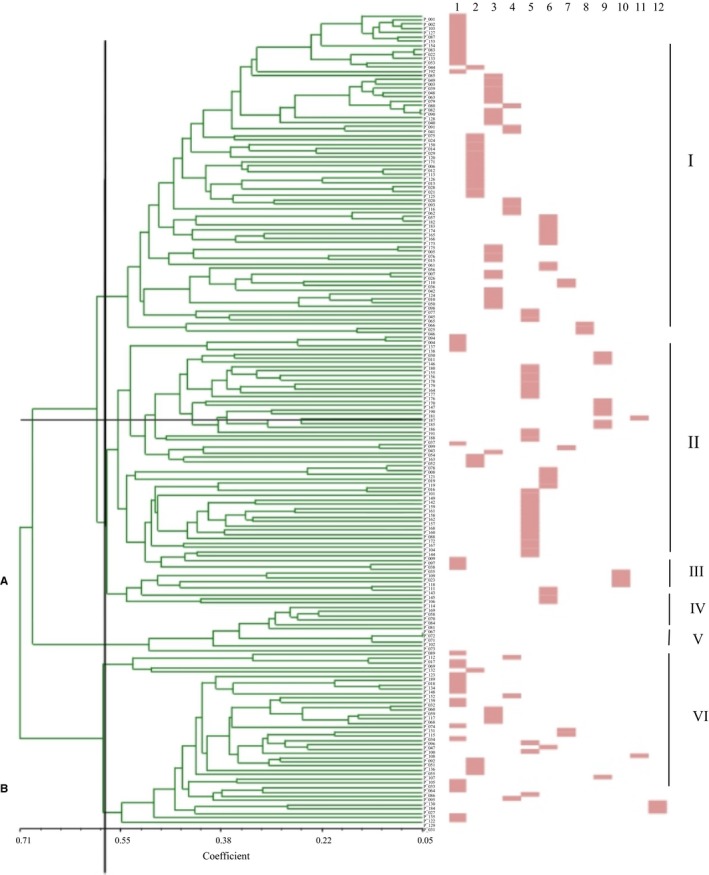
UPGMA dendrogram based on AFLP data and its relevance with 12 representative morphological characters of *Camellia reticulata* cultivars (the 12 types of flower features were showed in Table 5)

Based on the genetic and morphological data, an UPGMA dendrogram was constructed using NTSYSpc‐2.11F software (Hasani & Taghavi, 2014) to analyze the genetic structure combined with 12 representative flower characteristics in order to provide a comprehension evaluation of biodiversity in the *C. reticulata* accessions (Figure 4). Twelve morphological features were screened according to the importance and use frequency for cultivar classification by local informants. The phylogenetic tree resolved two major groups A and B with a similarity coefficient cutoff 0.545 (Figure 4). Group A is the largest and is composed of two subgroups (one that includes a large proportion of *C. reticulata* individuals that have double type flowers and the other subgroup separates all *C. japonica* individuals). Group B mainly consists of *C. reticulata* cultivars that have pink petals, double type flowers, and early maturity. *C. reticulata* samples in group A had a closer relationship to *C. japonica*, compared to *C. reticulata* samples in group B. This pattern is potentially due to the inclusion of many other *Camellia* species in the formation of *C. reticulata*'s polyploidization that have also contributed to its high genetic diversity. *C. japonica* as an outgroup in the phylogenetic tree was used to further discriminate the relationship of different cultivars of *C. reticulata*.

Group A and group B were further clustered into six subgroups with a similarity coefficient cutoff of 0.60, including four subgroups (I, II, III, and IV) in A and two subgroups (V and VI) in B (Figure 4). Cultivars with double type and dark flower petal color, including several samples collected from Tengchong were clustered together in subgroup I. Subgroup II clustered nearly all samples from Tengchong, which was one of the most representative of regional characteristics. Samples of *C. reticulata* in this cluster were distinctive for pink or white petal color, mostly semi‐double and a few double types. Some cultivars with single to semi‐double form collected from Laifeng Mountain and E'lu Mountain were placed in the subgroup III. All *C .japonica* samples were clustered together in subgroup IV and clearly separated from other subgroups of A. Subgroup V was the smallest subgroups comprised of only five cultivars from Kunming, Dali, Chuxiong, and Tengchong. This subgroup was distinctive for its special flower features. Subgroup VI had a complex cluster of cultivars including various petal colors and flower types.

### 3.7 Correspondence between genetic diversity and cultivar diversity of *C. reticulata*

**Figure 5:**
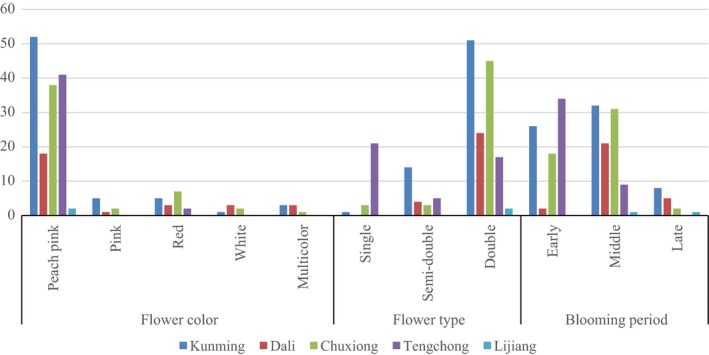
Distribution of three main morphological characteristics including flower color, flower type, and blooming period in five populations (Kunming, Dali, Chuxiong, Tengchong, and Lijiang)

**Figure 6:**
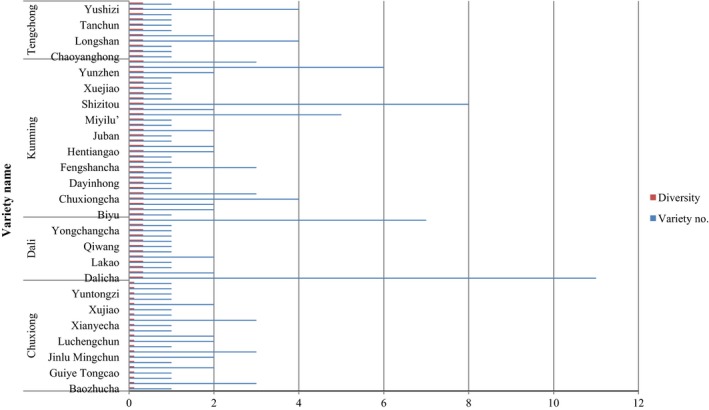
Correspondence between the genetic diversity and cultivar diversity of *Camellia reticulata*

Informants named a total of 75 cultivars of *C. reticulata* during our ethnobotanical survey and named these cultivars based on their morphological features, local customs, people's interests, and values. The population of *C. reticulata* in Kunming, Chuxiong, Dali, and Tengchong all has rich cultivar diversity. However, the populations from Kunming, Dali, and Tengchong have higher genetic diversity than the population of Chuxiong (Figure 6). The cultivars known as “Dalicha,” “Shizitou,” “Zipao,” “Zaotaohong,” and “Mudan” were found to be very common and popular in Yunnan with a genetic diversity of 0.3298, 0.3402, 0.3298, 0.3402, and 0.3402, respectively. As shown in Figure 5, this study did not have significant correspondence between genetic diversity and cultivar diversity of *C. reticulata* Except for the Chuxiong population, *C. reticulata* in the other three populations showed almost the same genetic diversity among different cultivars.

## 4 DISCUSSION

### 4.1 Genetic diversity and cultivar diversity of *Camellia reticulata*

*Camellia reticulata* is an endemic and religious tree restricted to Yunnan Province in south China. Our AFLP survey of that five natural populations of *C. reticulata* revealed a relatively high level of genetic diversity at the species level (*H* = 0.3578, *I* = 0.5369, PPL = 85.63%; Tables 1 and 2). Interestingly, a large proportion of genetic variation resided within populations (96.31%). By contrast, the genetic diversity of *C. reticulata* was relatively low between populations (3.69%) (Table 3). Findings showed that geographic distance among five populations was irrelevant to their genetic variations and distances (Figure 2 and Table 4). Therefore, geography is not the driving influence of the genetic diversity within populations of *C. reticulata*. Rather, the observed genetic diversity of *C. reticulata* is more likely due to the accumulation of different genotypes resulting from artificial hybridization facilitated by human cultivation practices and other cultural practices that serve to not only protect old cultivars but also to enhance new cultivars.

Comparison of the genetic data of *C. reticulata* with genetic data collected from populations of close congeners, *C. taliensis* (Zhao, Yang, Yang, Kato, & Luo, 2014) and *C. japonica* (Lin, Hu, Ni, Li, & Qiu, 2013), found no variation among wild populations. *C. taliensis* has a long domestication history as a tea tree widely distributed in Yunnan and has a relatively moderate to high level of overall gene diversity (*H*_s_ = 0.597) (Zhao et al., 2014). While no variation was found among wild populations in the AMOVA, most of the variation was detected within populations (70.6% within individuals and 16.5% among individuals within populations) rather than between populations (12.9% of variation was found among populations). Further, *C. japonica* is recognized to be widely distributed in China with relatively high genetic diversity (PPB: 90.1%; *H*_E_: 0.3414; *H*: 0.5013) (Lin et al., 2013). While a relatively high level of genetic differentiation among populations of *C. reticulata* was revealed by AMOVA (22.5%) by this study, relatively low genetic diversity existed within populations. This genetic trend is likely because of overexploitation, frequent human activities, and insufficient conservation management (Lin et al., 2013; Zhao et al., 2014).

Previous studies have supported that a long cultivation history, Buddhist practices, and other traditional cultural factors have contributed to the morphological diversity of *Camellia* trees (Xin et al., 2015). *C. reticulata* has a long history of use for worship and offerings in Buddhism in temples and altars in Yunnan Province by Bai, Yi, and other sociolinguistic groups (Xin et al., 2015). Many ancient trees of *C. reticulata* have been maintained for hundreds of years under the influence of Buddhism in temples and sacred areas in Yunnan (Xin et al., 2015). Other religions and beliefs adhered to the study areas are associated with conservation practices including those linked to nature worship, totemism, and ancestor worship (Long, Zhang, Pei, & Chen, 1999). For example, villages in Chuxiong of Yi Autonomous Prefecture build temples (called “Patron God Monastery”) (Yang & Sun, 2001) that consist of many varieties of *C. reticulata*. In addition, tree worship is one of the most important forms of worship in this area, especially for *C. reticulata* (Liu, Pei, & Chen, 2000). *C. reticulata* has played a role in the life of Yi of Chuxiong as a tribute to worship ancestors as well as for their farming practice (Lai, 2016). The Bai of Dali further regard *C. reticulata* a symbol of ancestral/nature spirits, cultural identity and status and give specific names for each cultivar based on different cultural symbols, meanings, and uses (Xin et al., 2015). For example, In Tengchong, a prized cultivar of *C. reticulata* named *Hong‐hua‐you‐cha* is considered distinct and highly valued for its seed oil hailed as “Oriental olive oil” that is widely used and commercialized for its high nutrition and medicinal values (Sahari, Ataii, & Hamedi, 2004). Findings from this study support that such cultural practices support rich genetic diversity and cultivar diversity of *C. reticulata* resources. Findings from our ethnobotanical surveys documented the conservation of old trees in temples within the study area and support that cultural beliefs and practices serve to protect old cultivars of *C. reticulata* to a large extent as well as influence their species diversity through natural and human selection and hybridization.

### 4.2 Population genetic structure and morphological structure

In our study, both Nei's genetic differentiation index among population (*G*_ST_ = 0.0536) and AMOVA (3.69%) values indicated no significant genetic differentiation among the studied populations. The low genetic differentiation exhibited by *C. reticulata* between populations can be explained by ancient economic and cultural exchange including the trade of natural resources such as tree and flower germplasm that has resulted in massive gene flow across ecosystems and populations in Yunnan. For example, the ancient Silk Road fostered economic and cultural exchange between Yunnan Province and its surrounding regions (Wang & Zhao, 2013). The map of the ancient South Silk Road (Figure 4) demonstrates that our study sites in Kunming, Chuxiong, Dali, and Tengchong were located in the important trade arteries and overlap with the distribution of *C. reticulata* in these areas. Except for Chuxiong (PPL = 28.94%; Table 2), all populations in this study were found to have a high genetic diversity at the population level including Kunming (PPL = 100%), Dali (PPL = 99.74%) and Tengchong (PPL = 99.74%). *C. reticulata* also has rich cultivar diversity in these four populations (Figure 6) with notable variation in flower characteristics (Figure 5). The main flower characteristic include peach pink color, double type petals, and a middle blooming period. These flower characteristics exist in the cultivars of Kunming, Dali, Chuxiong, and Tengchong and indicate that the morphological structure of *C. reticulata* in these four populations is not notably differentiated. This lack of differentiation may be due to economic exchange along the Silk Road that likely fostered the genetic diversity and cultivar diversity of *C. reticulata*. Traders exchanged the cultivars or other *Camellia* species from different places following their trade routes and play an important role in the high levels of genetic diversity within populations.

Although pollen of *C. reticulata* is dispersed by birds or insects naturally (Kunitake, Hasegawa, Miyashita, & Higuchi, 2004), the mountains in Yunnan where this species occur are distant from each other and have been recognized to result in genetic differentiation among populations (Ellstrand, 2014). Mountain geography has been likened to island geography in facilitating divergent evolution and fostering biodiversity (Winger & Bates, 2015). Gene flow through pollen migration by insects is most likely not a driving factor in the ecological evolution process of *C. reticulata* populations, but rather artificial propagation by humans that has been facilitated by cultural exchange and other cultural factors between different geographies.

## 5 CONCLUSION

This study highlights that *Camellia reticulata* resources have relatively high genetic diversity in Yunnan Province of southwestern China and that cultural factors may be a notable driving influence on fostering this diversity compared to geographic distance. AFLP was validated to be useful for examining the genetic evolution of *C. reticulata* as well as for elucidating genetic relationships of different *C. reticulata* cultivars by cluster analysis. This is the first study to provide evidence on the genetic diversity, structure, and differentiation within and among populations of *C. reticulata*. Traditional cultural practices and beliefs of different sociolinguistic groups in the study area of China have likely served an important role in the conservation and enhancement of *C. reticulata* diversity. We expect this study will be helpful for supporting biodiversity conservation, efforts for cultivar introduction, and further studies of *C. reticulata* and related species that are valued by different cultural groups.

## CONFLICT OF INTEREST

The authors declare that they have no conflict of interest.

## AUTHOR CONTRIBUTIONS

T.X. and W.J.H. wrote the manuscript as co‐first authors. C.L.L., J.D.R, and X.T. devised this study. T.X. collected the samples and conducted experiments. T.X. and W.J.H. analyzed the data. S.Z. and J.V.H were involved in the sampling and analyzing data. C.L.L., S.A., and W.J.H revised the manuscript.

## DATA ACCESSIBILITY

The AFLP data used to determine the genetic diversity of *Camellia* are furthermore provided within the Appendix S2.
