# Supplementary material for: Genetic diversity, population structure, and traditional culture of Camellia reticulata
Source: Ecol Evol. 2017 Sep 21;7(21):8915–26. doi: 10.1002/ece3.3340 (PMC5677478; doi:10.1002/ece3.3340)
Supplement: Supplementary file 1 [file ECE3-7-8915-s001.docx]

**Supplementary Information (Table S1-S5 and Fig. S1-S4)**

**Supplementary Tables:**

**Table  S1**The  sequence  of AFLP  primers  and  adapters  used  in  this  study

| Primer code | Primer name | sequence |
| --- | --- | --- |
| E1 | E-AAC | GACTGCGTACCAATTC+AAC |
| E2 | E-AGC | GACTGCGTACCAATTC+AGC |
| E3 | E-ACC | GACTGCGTACCAATTC+ACC |
| E4 | E-ACT | GACTGCGTACCAATTC+ACT |
| E5 | E-ACG | GACTGCGTACCAATTC+ACG |
| M1 | M-CAA | GATGAGTCCTGAGTAA+CAA |
| M2 | M-CAC | GATGAGTCCTGAGTAA+CAC |
| M3 | M-CTC | GATGAGTCCTGAGTAA+CTC |
| M4 | M-CAG | GATGAGTCCTGAGTAA+CAG |
| M14 | Msel-ad 14 | 5’-TACTCAGGACTCAT-3’ |
| M16 | Msel-ad 16 | 3’-GACGATGAGTCCTGAG-5’ |
| E17 | EcoRI-ad 17 | 5’-CTCGTAGACTGCGTACC-3’ |

**Table S2** AFLP  restriction  enzymes  digestion  system (total volume: 25μL)

| Components | Volume (μL) |
| --- | --- |
| 5×reaction buffer | 5 |
| MseI (10 U/μL) | 0.25 |
| EcoRI (15 U/μL) | 0.17 |
| Genomic DNA | 19.25 |
| ddH_2_O | 0.33 |

**Table S3** AFLP adaptor ligation solution

| Components | Volume (μL) |
| --- | --- |
| ddH_2_O | 19.96 |
| MseI – adaptor (50 pmol/μL) | 1 |
| EcoRI – adaptor (5 pmol/μL) | 1 |
| 1 mmol/L MgAc | 0.24 |
| 2 mmol/L KAc | 0.6 |
| 1 mmol/L Tris-HCl pH 7.5 | 0.24 |
| 10 mmol/L ATP | 0.96 |

**Table S4** PCR reaction system of AFLP selective amplification (total volume: 20μL)

| Components | Volume (μL) |
| --- | --- |
| ddH_2_O | 12.68 |
| 10 × PCR buffer | 2 |
| MseI - primer (1 μM) | 1 |
| EcoRI - primer (5 μM) | 1 |
| 20 mmol/LdNTP | 0.2 |
| Tag-polymerase (5 U/μL) | 0.12 |
| Preamplification product | 3 |

**Table S5** Total variance explained of *C. reticulata*

| Component | Initial Eigenvalues | | | Extraction Sums of Squared Loadings | | |
| --- | --- | --- | --- | --- | --- | --- |
|  | Total | % of Variance | Cumutive % | Total | % of Variance | Cumutive % |
| 1 | 116,448 | 61,613 | 61,613 | 116,448 | 61,613 | 61,613 |
| 2 | 18,437 | 9,755 | 71,368 | 18,437 | 9,755 | 71,368 |
| 3 | 13,488 | 7,137 | 78,504 | 13,488 | 7,137 | 78,504 |
| 4 | 5,786 | 3,062 | 81,566 | 5,786 | 3,062 | 81,566 |
| 5 | 2,875 | 1,521 | 83,087 | 2,875 | 1,521 | 83,087 |
| 6 | 2,441 | 1,291 | 84,379 | 2,441 | 1,291 | 84,379 |
| 7 | 1,700 | 0.899 | 85,278 | 1,700 | 0.899 | 85,278 |
| 8 | 1,551 | 0.821 | 86,099 | 1,551 | 0.821 | 86,099 |
| 9 | 1,435 | 0.759 | 86,858 | 1,435 | 0.759 | 86,858 |
| 10 | 1,145 | 0.606 | 87,464 | 1,145 | 0.606 | 87,464 |

**Supplementary Figures:**


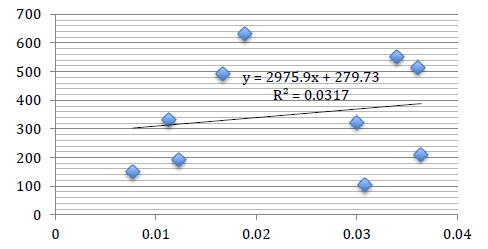


**Fig. S1** Correlation analysis of Nei’s unbiased genetic distance and geographic distance for five populations of *C. reticulata* (the unit of Y axis is km, the X axis represents for genetic distance)

**
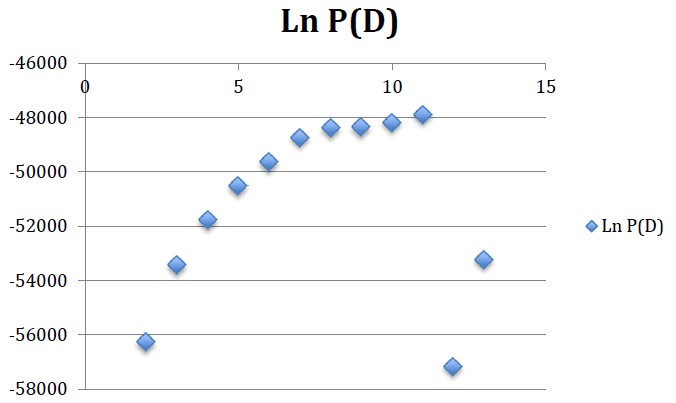
**

**Fig. S2** The panels show the log likelihood profiles mean Ln P(D) values based on K value from 2 to 14


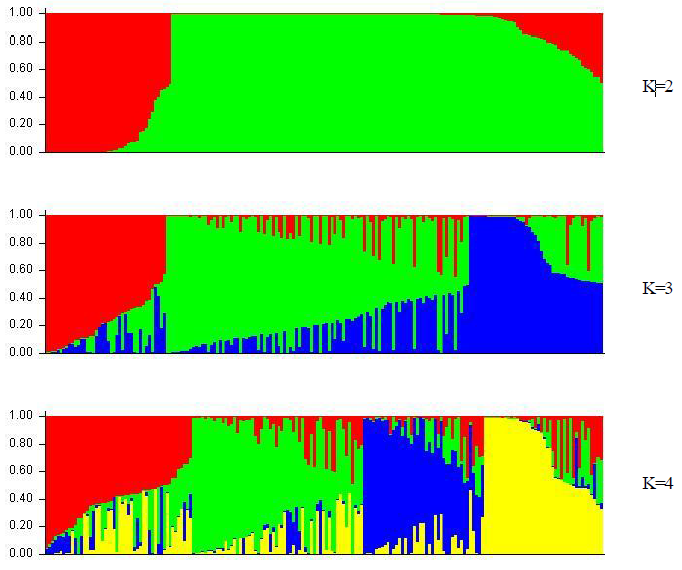


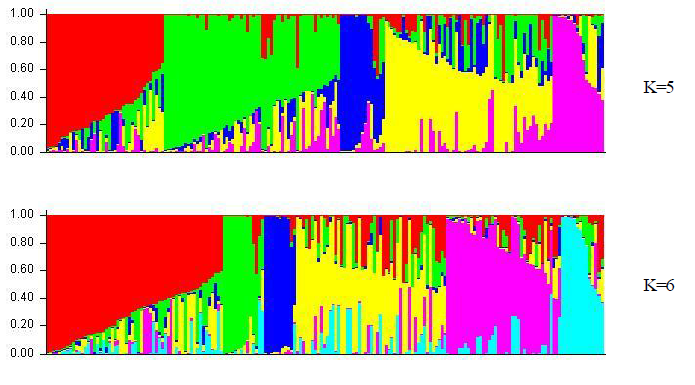


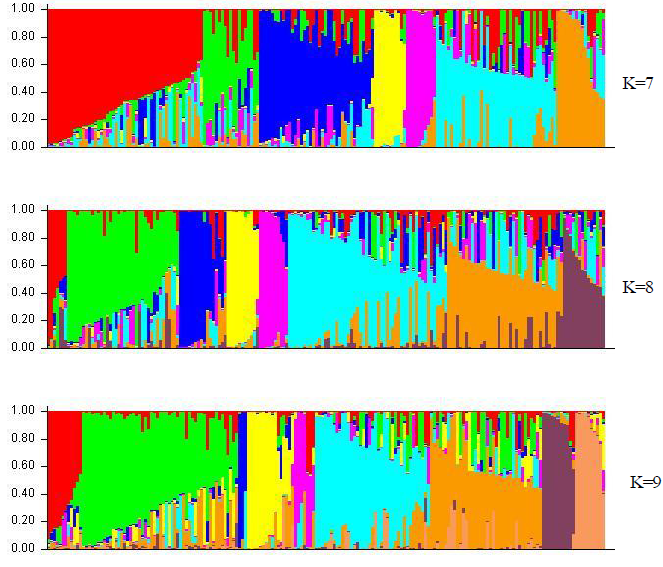


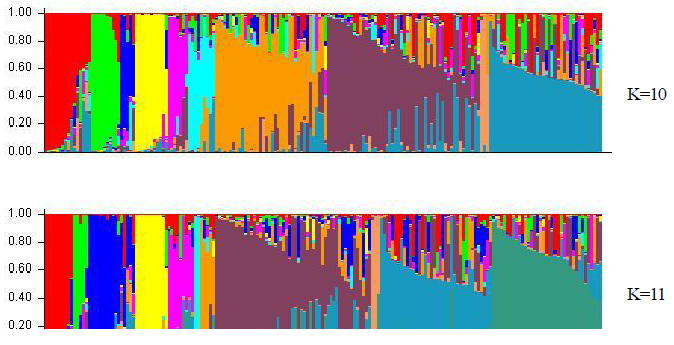


**Fig. S3** Population morphological structure model estimated by Q-single.


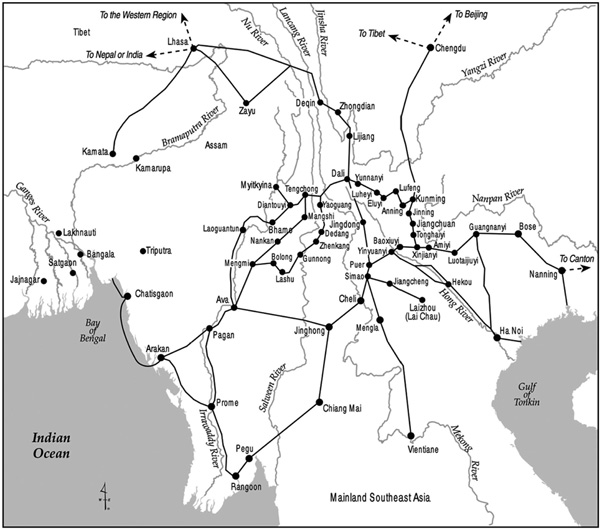


**Fig. S4** The Southwest Silk Road in the Yuan-Ming-Qing Period (Kunming, Tengchong, Dali and Lijian labeled with red asterisk) (A history of the communication between Yunnan and foreign countries) (Kunming: Yunnan Minzu Press, 1997).
